# Elucidation of the Biological Function and Early-Infection Cell Cycle Regulatory Mechanism of Avocado-Infecting *Colletotrichum fructicola*

**DOI:** 10.3390/foods15081295

**Published:** 2026-04-09

**Authors:** Sizhen Liu, Longhui Huang, Qianlong Sun, Yilong Man, Yangdan Yuan, Min Kuang, Yiyin Fan, Shankui Yuan, Yonghua Zhu, Xinqiu Tan

**Affiliations:** 1 Institute of Plant Protection, Hunan Academy of Agricultural Sciences, Yuelushan Laboratory, Changsha 410125, China; 2 Hunan Province Key Laboratory of Plant Functional Genomics and Developmental Regulation, College of Biology, Hunan University, Changsha 410082, China; 3 Longping Agricultural College, Hunan University, Changsha 410125, China; yonghuaz@outlook.com; 4 Key Laboratory of Pesticide Evaluation, Institute for the Control of Agrochemicals, Ministry of Agriculture and Rural Affairs, Beijing 100125, China; skyuan76@sina.com

**Keywords:** *Persea americana*, *Colletotrichum fructicola*, biological function, genetic transformation, cell cycle regulation

## Abstract

*Persea americana* (avocado) is a fruit rich in nutrients; however, its industry is facing major threats from pathogen infection. Here, we clearly identified *Colletotrichum fructicola* as the pathogen causing avocado diseases in Pu’er City, Yunnan Province. However, the biological characteristics, genetic transformation system, and early cell cycle regulation of this pathogen remained unclear. In this study, *C. fructicola* exhibited a maximum growth rate on complete medium (CM), with the conidial yield reaching 2 × 10^5^ conidia/mL after 24 h in liquid CM. Conidia of *C. fructicola* had nearly fully germinated at 4 h post-inoculation (hpi), with the appressorium formation rate exceeding 95% at 12 hpi. We also established a PEG-CaCl_2_-mediated genetic transformation system. The GFP-tagged transformants showed no significant differences in core biological function from the wild type. Using eGFP labeling, we visually elucidated the early cell cycle regulation of *C. fructicola*. Furthermore, cell cycle inhibitor assays demonstrated that *C. fructicola* conidial germination is independent of nuclear division and relies on cytoskeletal modulation, whereas appressorium formation and mycelial expansion require functional cell cycle regulation. This is probably the first study to systematically elucidate the cell cycle regulatory characteristics of *C. fructicola* isolated from avocado, and to successfully develop its genetic transformation system. These results provide important theoretical and technical support for the formulation of integrated control strategies against *C. fructicola*, as well as facilitating the sustainable development of the avocado industry.

## 1. Introduction

Avocado (*P. americana*), a nutrient-dense fruit rich in healthy fats, vitamins, and minerals, is widely cultivated in tropical and subtropical regions [1]. In recent decades, steadily growing global market demand has further solidified its importance in the tropical fruit sector [2]. However, with rapid industry development, the sustainable production of avocados is severely threatened by field and postharvest diseases [3,4]. Among these is anthracnose, which is one of the most destructive fungal diseases worldwide [3,5].

Anthracnose is a devastating disease caused by species of the genus *Colletotrichum*, and infects numerous crops globally, inflicting substantial economic losses on the agricultural and horticultural sectors [6,7]. Notably, *C. fructicola*, which is a key member of the *C*. *gloeosporioides* species complex, possesses exceptional host adaptability and inflicts widespread damage [8,9]. It can infect a variety of fruits such as mangoes, citrus, and longans, causing fruit rot and severely impairing fruit yield and quality [10,11,12]. *C*. *fructicola* is also capable of infecting forest trees such as oak and rubber, causing substantial economic losses [13,14]. It can also infect various precious medicinal plants, markedly reducing their quality [15,16].

At the molecular level, several virulence-related genes, transcription factors, and effector proteins governing growth, development, stress tolerance, and pathogenicity have been functionally characterized in *C. fructicola* isolates from other hosts [9,17,18]. For instance, the MdNIMIN2-MdNPR1 complex positively regulates plant immunity. In addition, *C. fructicola*’s CFEM (common fungal extracellular membrane) effector CfEC12 competitively binds to apple MdNIMIN2, interferes with the interaction between MdNIMIN2 and MdNPR1, weakens the defense response in apples, and thereby enhances the pathogenicity of the pathogen [19]. In *Camellia oleifera*-infecting *C. fructicola*, the histone acetyltransferase CfGcn5 positively regulates its growth, development, and pathogenicity [20], while the histone deacetylase CfSNT2 negatively regulates autophagy and is involved in the responses to host-derived reactive oxygen species (ROS) [21]. In pear-infecting *C. fructicola*, the xylanase CfXyn11A acts as a dual-function effector that degrades the plant cell wall to facilitate nutrient acquisition and induces a surge in reactive oxygen species and cell death in the non-host *Nicotiana benthamiana* [22]. To date, research on *C. fructicola* in avocado hosts has been limited to the isolation, identification, and morphological characteristics of this pathogen [8,23], with very few systematic studies on its pathogenic and infection mechanisms, or biological characteristics.

To successfully infect host plants, *Colletotrichum* species undergo a series of coordinated developmental processes, including conidial germination, appressorium formation, mycelial penetration, and intercellular spread [9]. These processes are precisely regulated by multiple signaling pathways, such as cell cycle control and cytoskeletal dynamics [24,25]. Accumulating evidence has confirmed that mitosis is critical for the growth, development, and pathogenicity of some phytopathogenic fungi [26]. For example, in *Magnaporthe oryzae*, hydroxyurea (HU, a G_1_/S phase inhibitor, targeting ribonucleotide reductase) inhibits mycelial growth [27]. Similarly, the inhibitory effect of increasing HU concentrations on the conidial germination of *M. oryzae* and *Fusarium oxysporum* was found to be dose-dependent [28,29]. The cytoskeletal system, composed of microtubules and actin filaments, serves as the structural basis for fungi, enabling them to maintain cell morphology, drive material transport, and mediate cell movement [30,31,32]. For instance, in *Pyricularia oryzae*, benomyl (a G_2_/M phase inhibitor, targeting microtubule assembly) and latrunculin A (Lat A, an actin polymerization inhibitor, targeting actin polymerization) disrupt the stability of hyphal polarity [33,34]. In *C. gloeosporioides* f. sp. *aeschynomene*, benomyl reduces the conidial germination rate and suppresses appressorium formation, whereas LatA completely abrogates conidial germination in this fungus [35]. However, cellular regulatory mechanisms differ markedly among fungal species, and the nature of cell cycle regulation in *C. fructicola* remains unknown.

In this study, the core biological traits of *C. fructicola* were systematically characterized and the regulatory roles of mitosis and cytoskeleton in its early critical infection stages were clarified. Furthermore, a stable genetic transformation system was successfully established for this strain, and using GFP-tagged transformants, the intercellular spread of *C. fructicola* within avocado host tissues was visually tracked. The findings not only deepen the understanding of the molecular interaction mechanism between *C. fructicola* and avocado, but also provide a solid scientific basis for postharvest control strategies against this pathogen.

## 2. Materials and Methods

### 2.1. Isolation and Purification of C. fructicola

According to Li’s method [36], in brief, tissue segments were excised from the disease-healthy junctions of Hass avocado fruit, placed in sterile centrifuge tubes, and subjected to surface sterilization sequentially with 70% ethanol for 1 min 30 seconds (s), followed by 2% sodium hypochlorite for 30 s. The sterilized segments were rinsed three times with sterile distilled water, then blotted dry on sterilized filter paper before being inoculated onto PDA plates. When a small number of mycelia emerged around the tissue segments, these newly grown mycelia were collected from the outermost edge and transferred to fresh PDA plates for subculture. Pure cultures of the pathogen were finally obtained using the single-conidium isolation method.

### 2.2. Strains and Culture Conditions

The wild-type (WT) strain was isolated and purified from the healthy-lesion tissue of Hass avocado fruits and named NY1. The strains were grown on PDA for routine maintenance and incubated at 28 °C. CM was used to prepare fresh mycelia for transformation and DNA and RNA extraction. TB3 solid medium and 5 × YEG liquid medium were used for transformant selection.

### 2.3. DNA Extraction, Sequence Acquisition, and Phylogenetic Tree Construction

Genomic DNA of *C. fructicola* was extracted using the CTAB (Solarbio, Beijing, China) method [37]. Using the genomic DNA of *C. fructicola* as the template, partial sequences of the internal transcribed spacer region (*ITS*), partial sequences of actin (*ACT*), and glyceraldehyde-3-phosphate dehydrogenase (*GAPDH*) genes were PCR-amplified (Takara, Dalian, China. All primers are listed in Table S1.

The purified PCR products were cloned following the manufacturer’s instructions for the pEASY^®^-T1 Vector (TransGen Biotech, Beijing, China). Positive clones were initially screened by colony PCR using the universal primers M13F and M13R. Plasmid DNA from PCR-positive colonies was submitted for Sanger sequencing.

The obtained sequences were assembled and subjected to BLAST 2.17.0 analysis in the NCBI database to confirm their identity. For phylogenetic analysis, the newly generated sequences for *ACT*, *ITS*, and *GAPDH* were concatenated. A dataset was constructed by aligning these concatenated sequences with 81 reference sequences from 11 species within the *Colletotrichum* genus and *Mucuna pruriens*, retrieved from the GenBank database (https://blast.ncbi.nlm.nih.gov/ accessed on 3 December 2025). Phylogenetic relationships were inferred using the maximum-likelihood method with a heuristic search, and the corresponding phylogenetic tree was reconstructed using MEGA 11 software. Bootstrap support values based on 1000 replications were calculated for the tree branches.

### 2.4. Morphological Observations of C. fructicola

Micro-morphological features of the *C. fructicola*, including conidial morphology, germ tube and appressorium formation, were observed using an inverted light microscope system from Zeiss Observer. A1 (Oberkochen, Germany), with concurrent image acquisition and quantitative data analysis.

### 2.5. Pathogenicity Assays

The pathogenicity of the isolated strains was verified via a pathogenicity assay following Koch’s postulates [38], using the conidial spray inoculation method. Briefly, purified strains cultured on PDA plates for 3 days (d) were inoculated into CM and incubated at 28 °C with shaking at 200 rpm for 3 d. Conidia were harvested by filtration through two layers of sterile Miracloth, and the resulting conidial suspension was washed with sterile water, followed by centrifugation at 8000 rpm for 8 min. This washing-centrifugation procedure was repeated twice, and the final conidial pellet was resuspended in sterile water.

A 5 mL aliquot of the conidial suspension (2 × 10^6^ conidia/mL) was uniformly sprayed onto the sterilized Hass healthy avocado fruit surfaces. In the control group, fruits were sprayed with sterile water instead. All treated fruits were placed in sterile Petri dishes, maintained at 80% relative humidity, and incubated in darkness at 28 °C. To fulfill Koch’s postulates, the pathogen was re-isolated and purified from the symptomatic tissues of inoculated fruits.

### 2.6. Mycelial Growth Assays

Mycelia plugs (5 × 5 mm) of WT were incubated on PDA, OM, SDC, V8, and CM agar plates, separately. At 1–6 days post-inoculation (dpi), the colony diameters were measured and statistically analyzed. All media formulations are listed in Table S2.

WT was used after single-spore isolation and three successive rounds of activation.

### 2.7. Mycelial Conidia Production Assays

Mycelial plugs of uniform quantity and size were incubated in liquid CM with shaking at 200 rpm and 28 °C for 3 d. The conidial concentration was quantified at 24-hour (h) intervals using a hemocytometer under a light microscope.

### 2.8. Conidial Germination and Appressorium Formation Assays

The purified strains grown for 3 d on PDA plates were incubated in CM at 28 °C for 3 d with shaking at 200 rpm. An amount of 20 µL of 2 × 10^5^ conidia/mL of conidial suspension was inoculated on the hydrophobic surface, and glass slides were fixed onto filter paper and placed in 150 mm Petri dishes containing 8 mL sterile water at 28 °C in the dark. The extent of conidial germination and appressorium formation was observed and counted under a microscope at 2 h intervals.

A total of 20 µL of 2 × 10^5^ conidia/mL conidial suspension was inoculated on cellophane with three different concentrations of hydroxyurea (HU, 15 mM, 30 mM, and 60 mM), benomyl (2.5 µg/mL, 5 µg/mL, and 10 µg/mL), and Latrunculin A (LatA, 2.5 µm, 5 µm, and 10 µm). The samples were placed into 150 mm Petri dishes containing 8 mL of sterile water at 28 °C in the dark. Conidial germination and appressorium formation were observed under a microscope at 4 hpi and 6 hpi, respectively.

In each experiment, 200 conidia were counted, and the conidial germination rate and appressorium formation rate were calculated.

### 2.9. GFP-Tagged Genetic Transformation Assays

#### 2.9.1. Hygromycin B Sensitivity Assay

Sterile mycelial plugs were excised from the actively growing margin of the colonies and transferred onto PDA plates supplemented with hygromycin B at a series of gradient concentrations (0, 20, 40, 60, 80, and 100 mg/L). All plates were incubated in a constant-temperature incubator at 28 °C for 5 d, after which mycelial growth was observed and recorded.

#### 2.9.2. Protoplast Preparation

For the enzyme solution, 0.3 g of lysing enzyme (Sigma, St. Louis, MI, USA SLBJ0553V) was dissolved completely in 0.7 M NaCl at room temperature, adjusted to a final volume of 30 mL, and filter-sterilized using a 0.22 µm membrane filter.

For the 1 × STC buffer, 100 g sucrose, 3.0285 g Tris-HCl (50 mM, pH 8.0), and 2.7745 g CaCl_2_ (50 mM) were dissolved in dd H_2_O to a final volume of 500 mL, and autoclaved at 120 °C for 20 min.

Fresh mycelial plugs were excised from single-conidium-derived colonies of *C. fructicola* and minced thoroughly. They were then transferred into 5 × YEG liquid medium. The cultures were incubated at 28 °C with shaking at 80 rpm for 10 h. Mycelia were harvested by filtration through three layers of sterile Miracloth, rinsed five times with sterile water, and blotted dry with sterile filter paper to remove residual moisture.

The harvested mycelia were transferred to a 50 mL sterile centrifuge tube containing 30 mL of enzyme digestion solution for enzymatic hydrolysis. The tube was incubated horizontally on a rotary shaker at 30 °C and 60 rpm for 2 h. The undigested mycelial debris was removed by filtration through three layers of sterile Miracloth, and the filtrate was collected in a new sterile centrifuge tube. The filtrate was centrifuged at 3500 rpm and 4 °C for 5 min. The supernatant was carefully discarded, and the protoplast pellet was gently resuspended in an appropriate volume of STC solution. The protoplast concentration was quantified, and the suspension was adjusted to a final concentration of 1 × 10^8^ protoplasts/mL.

#### 2.9.3. PEG-CaCl_2_-Mediated Protoplast Transformation

For the PTC buffer, 120 g PEG4000 (Polyethylene Glycol 4000) was weighed and completely dissolved in 1 × STC buffer. It was then, adjusted to a final volume of 1 L and autoclaved at 120 °C for 20 min.

The SK1044 plasmid was used as the template, and specific primers were designed to amplify the DNA fragment (approximately 3000 bp) harboring the T-DNA left and right borders, *eGFP* gene, and hygromycin B resistance gene (*Hph*). The PCR product was purified and used for subsequent genetic transformation.

A total of 2 µg of the purified PCR product was added to 150 µL of *C. fructicola* protoplast suspension, and the mixture was incubated at room temperature for 25 min. Subsequently, 500 µL of PTC solution was added using a cut-off pipette tip (to avoid protoplast damage) and the mixture was gently mixed. After standing for another 15 min, an additional 500 µL of PTC solution was added, gently mixed, and the mixture was incubated for a further 10 min.

Finally, the protoplast–PTC mixture was added to 10 mL of molten TB3 medium (cooled to 50 °C) supplemented with 20 mg/L hygromycin B, gently mixed, and poured into sterile Petri dishes. The dishes were incubated in the dark at 28 °C for 24 h, then overlaid with 10 mL of TB3 medium containing 40 mg/L hygromycin B, and incubation in the dark continued. Once transformants emerged, single colonies were removed with sterile toothpicks and transferred to PDA medium supplemented with 40 mg/L hygromycin B, for screening of positive transformants.

#### 2.9.4. GFP-Tagged Transformant Verification

Genomic DNA was extracted from both the mycelia of NY1 and transformant strains, respectively, and used as a template for polymerase chain reaction (PCR) amplification with GFP-specific primers. The resulting PCR products were subjected to detection via 1% agarose gel electrophoresis.

Mycelia and conidia of the PCR-verified transformants were separately prepared on microscopic slides and observed using a Nikon C2 (Tokyo, Japan) laser scanning confocal microscope. Both the mycelia and conidia of successfully transformed strains exhibited strong green fluorescence signals.

### 2.10. Verification of Biological Functions for GFP-Tagged Transformants

Sterile 5 mm × 5 mm mycelial plugs were excised from the fresh colony transformants and WT. The plugs were separately transferred onto five different solid media, namely PDA, OM, V8, SDC, and CM. All plates were incubated in a constant-temperature incubator at 28 °C, and the mycelial colony diameter was measured periodically using a vernier caliper.

For conidial production analysis, sterile 5 mm × 5 mm mycelial plugs were similarly excised from the fresh colony margins of GFP-tagged transformants and WT. The plugs were minced into small fragments with a sterile scalpel, transferred into CM, and incubated at 28 °C with shaking at 200 rpm for 3 d. The conidial concentration was quantified using a hemocytometer under a light microscope at 24 h intervals.

Both the WT and GFP-tagged strains were used after single-spore isolation and three successive rounds of activation.

### 2.11. Pathogenicity Assays of GFP-Tagged Transformants

A total of 5 mL of conidial suspension (2 × 10^6^ conidia/mL) from GFP-tagged transformants and WT was separately evenly onto the surface of surface-sterilized and artificially wounded Hass avocados, respectively. In the control group, fruits were sprayed with sterile water instead. All treated fruits were placed in sterile Petri dishes and maintained under moist conditions, followed by incubation in the dark at 28 °C in a constant-temperature incubator. Disease symptoms were observed and recorded starting at 2 dpi.

### 2.12. Intercellular Spread of C. fructicola During Plant Infection Assays

A total of 20 μL of 2 × 10^5^ conidia/mL conidial suspension derived from GFP-tagged transformants was inoculated onto an onion epidermal cell. The inoculated peels were placed in, and glass slides were fixed onto filter paper and placed into 150 mm Petri dishes containing 8 mL of sterile water at 28 °C in the dark. Dynamic observations of the infection and intercellular colonization process of *C. fructicola* were performed using a laser scanning confocal microscope at both 24 hpi and 48 hpi.

### 2.13. Effect of Cell Cycle Inhibitors on C. fructicola Intercellular Spread Assays

A 20 μL aliquot of the conidial suspension (2 × 10^5^ conidia/mL) from GFP-tagged transformants was inoculated onto an onion epidermal cell. The inoculated peels were then placed into 150 mm sterile Petri dishes containing 8 mL of sterile water supplemented with different concentrations of 30 mM HU, 5 µg/mL benomyl, and 10 µg/mL Lat A, followed by incubation in the darkness at 28 °C under moist conditions. The intercellular expansion of *C. fructicola* mycelia was observed and recorded using a laser scanning confocal microscope at both 24 hpi and 48 hpi.

### 2.14. Statistical Analysis

All experiments were repeated three times using three independent biological replicates. The data were expressed as means ± SEs. Significant differences (* *p* < 0.05) between the treatments were determined according to the Tukey−Kramer test for evaluating the difference between two groups. All statistical analyses were performed using GraphPad Prism 8.0 software.

## 3. Results

### 3.1. Pathogen Symptom Characterization

**Figure 1:**
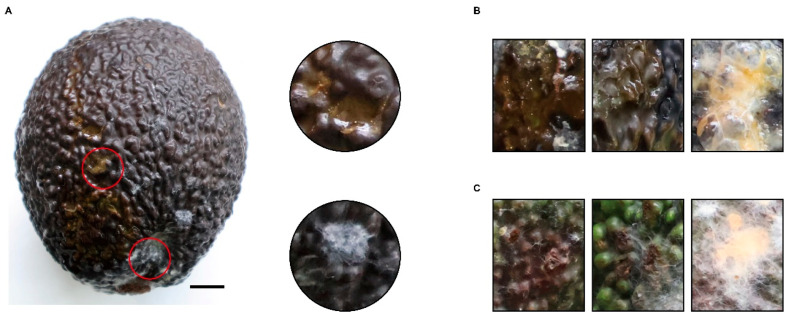
Symptoms of avocado fruit infected by the pathogen. (**A**) Sample of diseased fruit. The scale bar represents 1 cm. (**B**,**C**) Typical symptoms of diseased fruits.

Initially, light brown, slightly sunken lesions developed on the epidermis of infected avocado fruits (Figure 1A). As the infection advanced, these primary lesions coalesced and expanded into extensive dark brown to near-black necrotic lesions (Figure 1A,B). These symptomatic regions were accompanied by epidermal wrinkling and pronounced wet rot symptoms at the pathogen colonization sites (Figure 1B). Under high-temperature and humidity conditions, the lesion surfaces became covered with white colonies of the pathogen, from which grayish-yellow to orange-red conidial masses were subsequently produced (Figure 1A,C). The pathogenic characteristics of this pathogen are similar to those of *Colletotrichum* pathogens [3].

### 3.2. Mycelial and Conidial Morphology of the Pathogen

**Figure 2:**
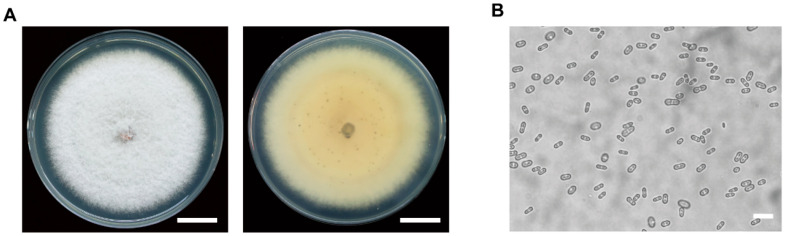
Colony and conidia morphology of the avocado-infecting pathogen. (**A**) Colony on PDA medium at 10 days post-inoculation (dpi). The scalebar represents 2 cm. (**B**) Conidial morphology under a microscopic observation. The scale bar represents 200 µm.

The NY1 strains were isolated from the healthy-diseased tissue interface of anthracnose-infected avocado fruits. On PDA medium, the NY1 strain exhibited a distinct colony morphology that varied with the culture stage. In the early culture stage, colonies were predominantly white with dense, flocculent hyphae. With prolonged incubation, orange-yellow pigments gradually formed in the colony center, accompanied by fine black spots (Figure 2A). The conidia of NY1 were ellipsoidal and septate, measuring 55.385–172.216 µm in length and 21.724–87.269 µm in width (n = 200) (Figure 2B). The morphological characteristics are similar to published descriptions of *C. fructicola* [39].

### 3.3. Identification of Avocado Pathogen and Phylogenetic Tree Construction

To further confirm the species classification of the pathogenic fungus, we amplified partial gene sequences of the internal transcribed spacer region (ITS), actin (ACT), and glyceraldehyde-3-phosphate dehydrogenase (GAPDH) genes using genomic DNA from NY1 as the template (Figure S1). BLAST analysis showed 100%, 100%, and 100% sequence homology with *C. fructicola* reference sequences OQ184898, MT424615, and MN075663, respectively. The sequences of these three genes were submitted to GenBank; and the GenBank accession numbers are PX659873 (ITS), PX683720 (ACT), and PX683721 (GAPDH), respectively.

**Figure 3:**
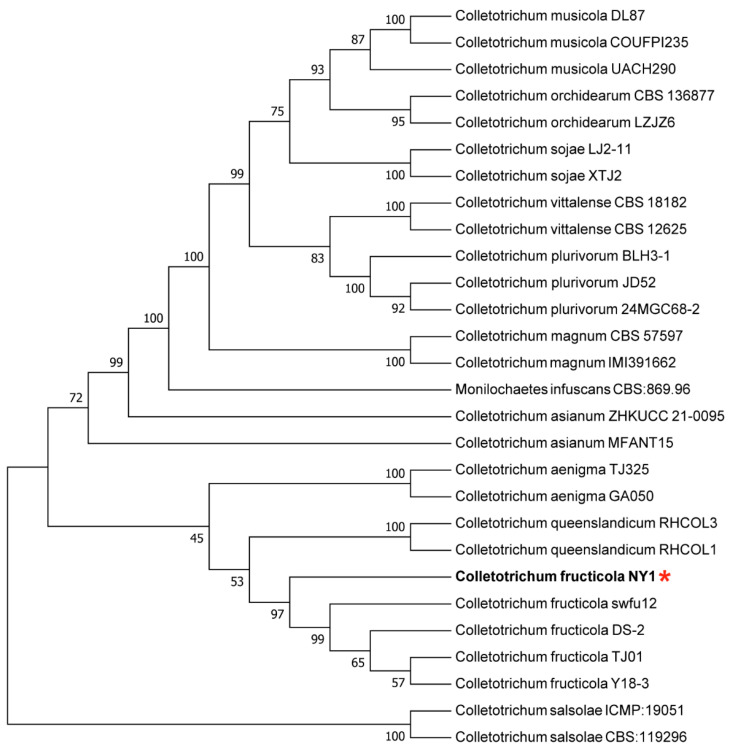
Maximum likelihood tree of *C. fructicola* constructed from combined ITS, ACT, and GAPDH gene sequence data. The numbers above the branches show bootstrap support values inferred from maximum likelihood. Bootstrap support values based on 1000 replications were calculated for the tree branches. The newly obtained *C. fructicola* strain NY1 in this study is marked with a red asterisk.

Phylogenetic analysis was conducted using MEGA 11 with the maximum likelihood method and concatenated sequences were aligned against 81 reference sequences representing 12 taxa from GenBank (Table 1). Phylogenetic analysis revealed that strain NY1 clustered with reported *C. fructicola* reference strains in a distinct cluster, indicating high sequence homology with *C. fructicola* species (Figure 3). Combined with the observed morphological characteristics and multi-locus molecular identification, the pathogen strain was ultimately identified as *C. fructicola*.

### 3.4. C. fructicola NY1 as the Pathogen of the Avocado Anthracnose

**Figure 4:**
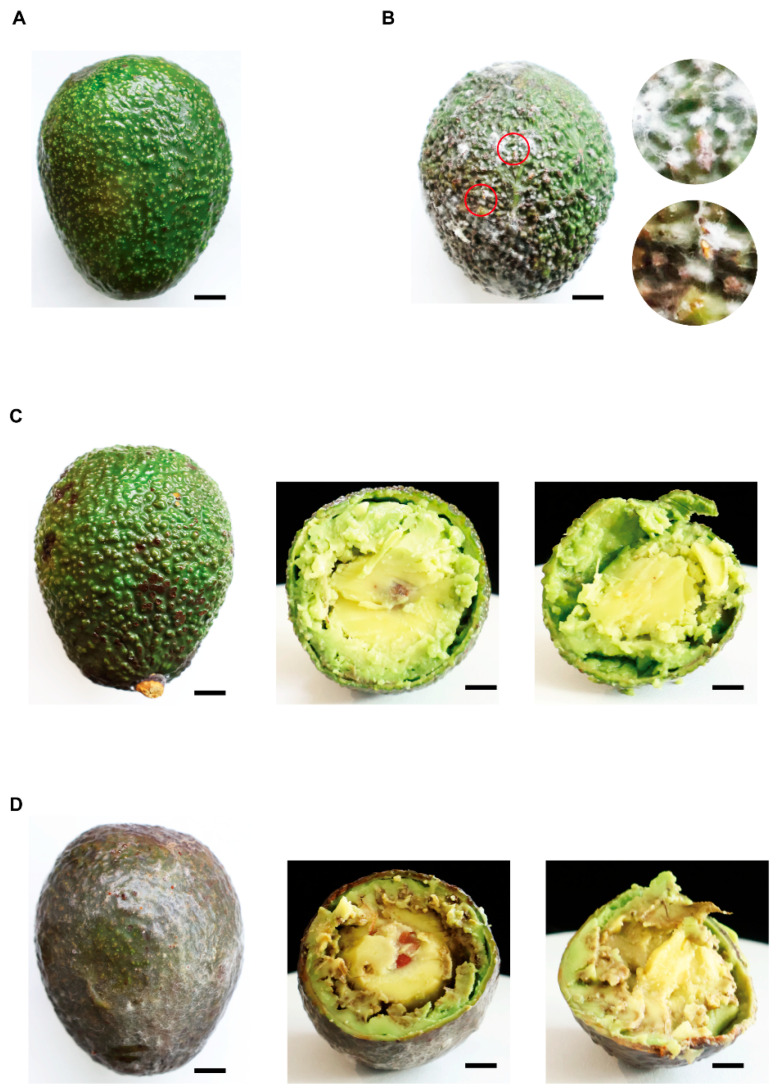
Pathogenicity re-test of the Pathogen. (**A**) Avocado fruits were inoculated with sterile water at 10 dpi. (**B**) Symptoms and magnified views of avocado fruits inoculated with *C*. *fructicola* 10 dpi. (**C**) Longitudinal sections of avocado fruits inoculated with sterile water. (**D**) Longitudinal sections of avocado fruits inoculated with *C*. *fructicola*. All scale bars represent 1 cm in Figure 4.

To confirm that NY1 is the causal agent of field avocado anthracnose, Koch’s postulates were verified via spray inoculation with conidia. At 10 days post-inoculation (dpi), the control fruits sprayed with sterile water remained asymptomatic and retained normal lime-green coloration (Figure 4A,C). In contrast, white mycelia, orange-red conidial masses, and distinct brown necrotic lesions developed on the surface of the NY1-inoculated fruits (Figure 4B). Internally, the fruit flesh developed black-brown soft rot when sectioned (Figure 4D). The strain was reisolated from the re-inoculated fruits, and the morphological and molecular characterization was consistent with that of the *C. fructicola* NY1 strain. Thus, the results confirmed that NY1 is the pathogen responsible for the avocado anthracnose observed in the field.

### 3.5. Mycelial Growth and Conidia Production Rates of C. fructicola

**Figure 5:**
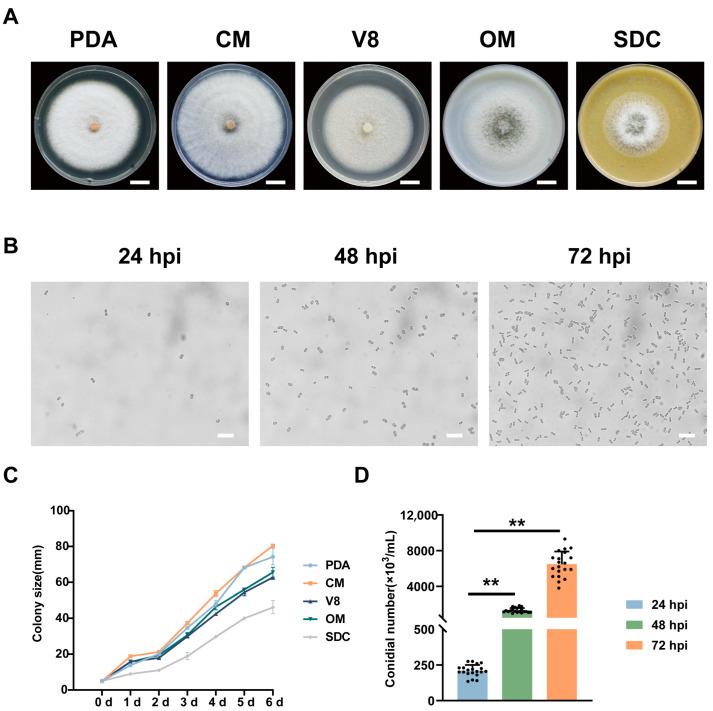
Mycelial growth and conidial production rates of *C. fructicola*. (**A**) Colony formation of *C. fructicola* on PDA, CM, OM, V8, and SDC media at 6 dpi, respectively. The scale bar represents 2.5 cm. (**B**) Conidial yield under microscopic observation in CM at 24 hours (h), 48 h, and 72 h. The scale bar represents 200 µm. (**C**) Growth curves of *C. fructicola* cultured on five media at 6 dpi (n = 3 independent experiments). (**D**) Statistical analysis of conidial yield in (**B**) (n = 3 independent experiments). The above data are means ± SEs. Means with different asterisks are significantly different, as determined by the Tukey−Kramer test (** *p* < 0.01).

To evaluate the mycelial growth and sporulation capacity of *C. fructicola* strain NY1 (wild type, WT), mycelial plugs were inoculated onto five solid media (PDA, CM, V8, OM, SDC). At 6 dpi, the colony diameters of *C. fructicola* exhibited significant differences in the growth rates on different media, with the fastest radial growth observed on CM, followed by PDA, OM, V8, and SDC (Figure 5A,C).

Mycelial plugs of the same size from the WT were inoculated into liquid CM for shaking culture. After 24 hours (h), the conidia yield reached 2 × 10^5^ conidia/mL, increasing to 1 × 10^6^ conidia/mL at 48 h and 6 × 10^6^ conidia/mL at 72 h (Figure 5B,D). This indicates *C. fructicola*’s robust conidia production capacity.

### 3.6. Conidial Germination and Appressorium Formation of C. fructicola

**Figure 6:**
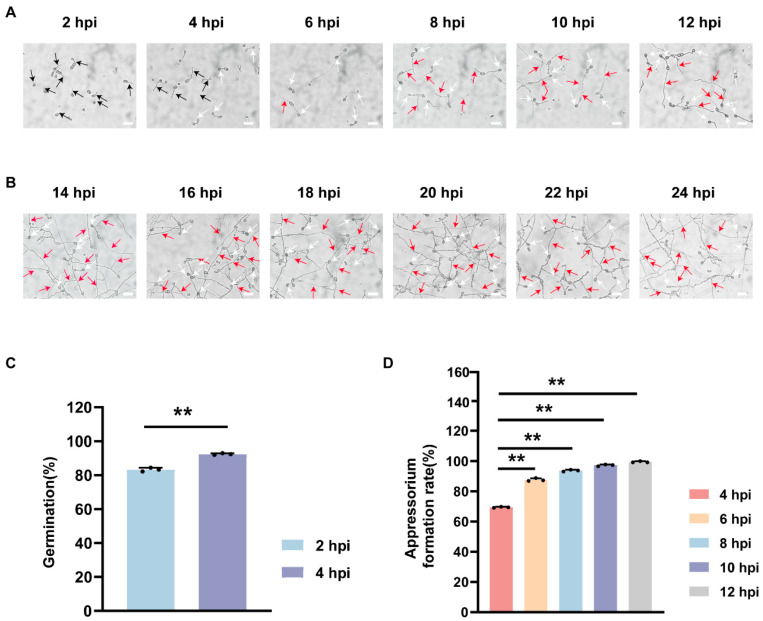
Conidial germination and appressorium formation of *C. fructicola*. (**A**) Process of conidial germination and appressorium formation in *C. fructicola* on hydrophobic surfaces from 2 to 12 hpi. Black arrows indicate germ tubes, White arrows indicate appressorium, and red arrows indicate primary mycelia. The scale bar represents 200 µm. (**B**) Process of appressorium formation of *C. fructicola* on hydrophobic surfaces from 14 to 24 hpi. White arrows indicate appressorium, and red arrows indicate primary mycelia. The scale bar represents 200 µm. (**C**) Quantification of conidial germination rate of *C. fructicola* at 2 and 4 hpi (n = 3 independent experiments). The above data are means ± SEs. Means with different asterisks are significantly different, as determined by the Tukey−Kramer test (** *p* < 0.01). (**D**) Quantification of appressorium formation rate of *C. fructicola* from 4 to 12 hpi (n = 3 independent experiments). The above data are means ± SEs. Means with different asterisks are significantly different, as determined by the Tukey−Kramer test (** *p* < 0.01).

To characterize the temporal aspects of conidial germination and appressorium formation in the WT, 20 µL of a 2 × 10^5^ conidia/mL suspension was inoculated onto the hydrophobic surfaces. The conidial germination rate reached 83% at 2 h post-inoculation (hpi) and exceeded 92% by 4 hpi in the WT (Figure 6A,C). Meanwhile, the WT appressorium formation rate reached 68% at 4 hpi and peaked at 99% at 12 hpi (Figure 6A,D). At 6 hpi, conidia of the WT germinated continuously on the side opposite to appressorium formation, producing primary hyphae. (Figure 6A,B). These results indicate rapid conidial germination and appressorium formation in the WT, accompanied by primary hyphal extension from the non-appressorium pole of germinating conidia.

### 3.7. Establishment of a GFP-Tagged Genetic Transformation System for C. fructicola

To facilitate dynamic observations of infection process of *C. fructicola* and provide technical support for gene knockout-mediated functional studies, it is imperative to construct a stable genetic system for this fungus. Therefore, we first obtained GFP-tagged transformants through hygromycin resistance-based screening. For optimal transformant screening, the mycelial plugs of the WT were inoculated onto hygromycin B-supplemented PDA (0–100 mg/L), with colony growth evaluated at 5 hpi. Colony growth was significantly inhibited at 20–100 mg/L (Figure S2A). Specifically, it was markedly reduced at 20 mg/L and nearly eliminated at 40 mg/L (Figure S2A). Thus, 40 mg/L was selected as the screening concentration for subsequent transformations.

**Figure 7:**
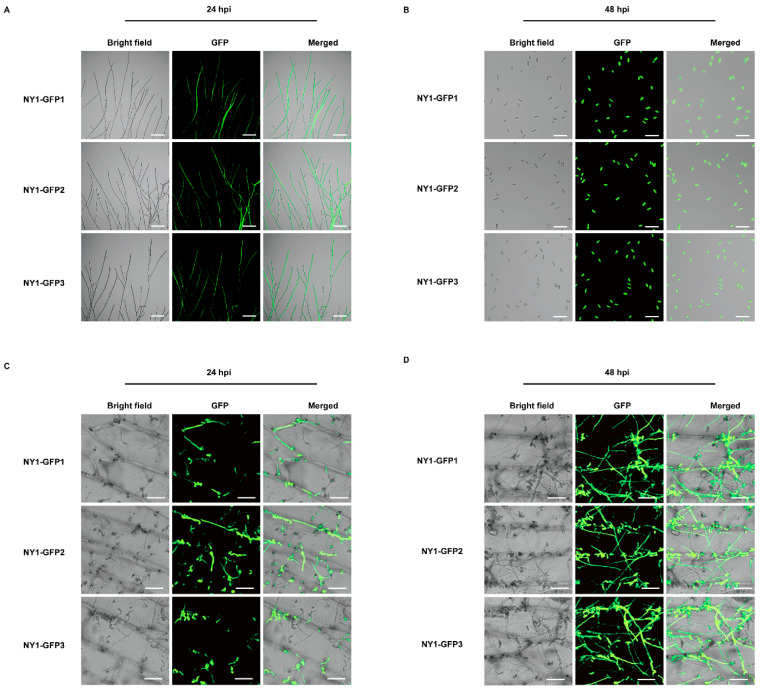
Early intercellular infection of conidia from GFP-tagged transformants on onion epidermal cells. (**A**) Mycelia of GFP-tagged transformants under a laser confocal microscope. The scale bar represents 100 µm. (**B**) Conidia of GFP-tagged transformants under a laser confocal microscopy. The scale bar represents 100 µm. (**C**) Intercellular infection status of conidia from GFP-tagged transformants at 24 hpi on onion epidermis. Scale bar represents 50 µm. (**D**) Intercellular infection status of conidia from GFP-tagged transformants at 48 hpi on onion epidermis. The scale represents 50 µm.

*C. fructicola* protoplasts were then transformed with expression vectors harboring the hygromycin B resistance and *eGFP* genes. Eight randomly selected resistant transformants were verified by PCR with GFP-specific primers, and agarose gel electrophoresis confirmed target-sized amplicons in all (Figure S2B). Three randomly selected GFP-tagged transformants were subjected to fluorescence detection via a laser scanning confocal microscope. Subsequently, stable green fluorescence was detected in their mycelia and conidia. The three GFP-tagged transformants were designated NY1-GFP1, NY1-GFP2, and NY1-GFP3 (Figure 7A,B). These results confirm the successful genomic integration and functional expression of the GFP sequence in *C. fructicola*.

### 3.8. Comparison of Core Biological Functions Between C. fructicola and GFP-Tagged Trans formants

To determine whether the biological characteristics of the three GFP-tagged transformants differed from those of *C. fructicola*, mycelial growth was evaluated by measuring the colony diameters of NY1, NY1-GFP1, NY1-GFP2, and NY1-GFP3. At 5 dpi, there were no significant differences in mycelial growth between these GFP-tagged transformants and NY1 (Figure S3).

Subsequently, mycelial plugs from NY1, NY1-GFP1, NY1-GFP2, and NY1-GFP3, of uniform size and equal growth stage, were inoculated into liquid CM for shake culturing. Conidial yield was quantified at 24, 48, and 72 hpi. At all three time points, there were no significant differences in conidial yield compared with NY1 (Figure S4).

Then, to compare the pathogenicity of GFP-tagged transformants with NY1, conidial suspensions of both were sprayed onto wounded avocado fruit surfaces. At 15 dpi, the control fruits remained green and healthy (Figure S5A), while inoculated NY1 fruits developed brown lesions with white mycelia, pink conidial masses, and yellowish-brown exudate (Figure S5B). Moreover, cross-sections revealed brown, rotten flesh beneath lesions (Figure S5B), and fruits infected by these GFP-tagged transformants developed similar symptoms to those infected by NY1 (Figure S5C–E).

Finally, laser confocal microscopy observations showed the labeled *C. fructicola* germinated and formed primary hyphae in the onion epidermis at 24 hpi (Figure 7C). Then, at 48 hpi, the labeled hyphae further invaded and spread into the intercellular spaces of the onion epidermal tissues, enabling clear visualization of the pathogen’s intercellular dissemination in host tissues (Figure 7D). These results confirmed that GFP labeling effectively visualized the intercellular expansion of *C. fructicola*.

All in all, these findings indicate that the core biological function of *C. fructicola* was maintained in the GFP-tagged transformants.

### 3.9. Investigation of the Dependence of Conidial Germination on Nuclear Division and the Cytoskeleton of C. fructicola

**Figure 8:**
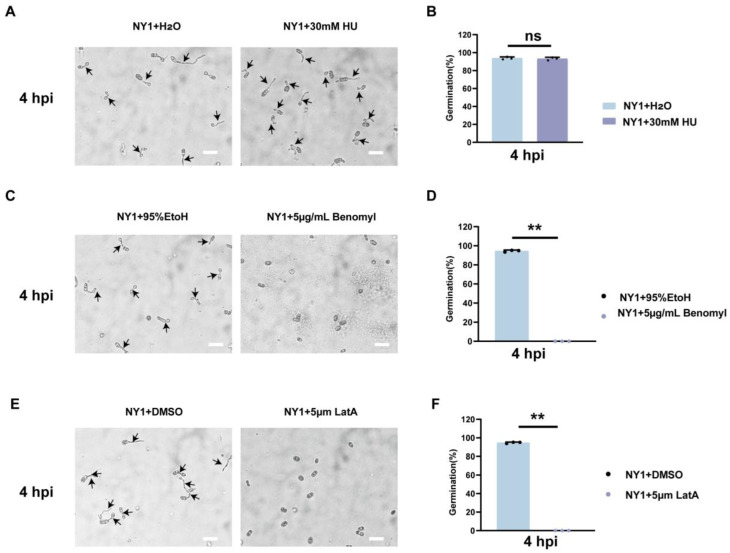
Effect of cytoskeleton on conidial germination of *C. fructicola*. (**A**) Conidial germination of *C. fructicola* treated with 30 mM hydroxyurea (HU) at 4 hpi. Black arrows indicate germ tubes. The scale bar represents 200 µm. (**B**) Statistical analysis of conidia germination in (**A**) (n = 3 independent experiments). The above data are means ± SEs. Means with different asterisks are significantly different, as determined by the Tukey−Kramer tes. ns, not significant. (**C**) Conidial germination of *C. fructicola* treated with 5 μg/mL benomyl at 4 hpi. Black arrows indicate germ tubes. The scale represents 200 µm. (**D**) Statistical analysis of conidial germination in (**C**) (n = 3 independent experiments). The above data are means ± SEs. Means with different asterisks are significantly different, as determined by the Tukey−Kramer test (** *p* < 0.01). (**E**) Conidial germination of *C. fructicola* treated with 5 μm latrunculin A (lat A) at 4 hpi. Black arrows indicate germ tubes. The scale bar represents 200 µm. (**F**) Statistical analysis of conidial germination in (**E**) (n = 3 independent experiments). The above data are means ± SEs. Means with different asterisks are significantly different, as determined by the Tukey−Kramer test (** *p* < 0.01).

To investigate the dependence of *C. fructicola* conidial germination on nuclear division, the hydroxyurea (HU, a G1/S phase nuclear division inhibitor), benomyl (a G2/M phase mitosis inhibitor), and LatA (an actin polymerization inhibitor) were separately added to the conidial suspension (2 × 10^5^ conidia/mL). At 6 hpi, HU-treated conidia of NY1 germinated normally, with no significant difference in germination rate compared with the control group (Figure 8A,B). In contrast, benomyl and LatA completely inhibited the germination of NY1 conidia at all tested concentrations (Figure 8C–F). However, none of the three cell cycle inhibitors exhibited dose dependence (Figure S6). These findings demonstrate that conidial germination relies on cytoskeletal integrity rather than nuclear division.

### 3.10. Exploration of the Relationship Between Appressorium Normal Formation and the Complete Cell Cycle of C. fructicola

**Figure 9:**
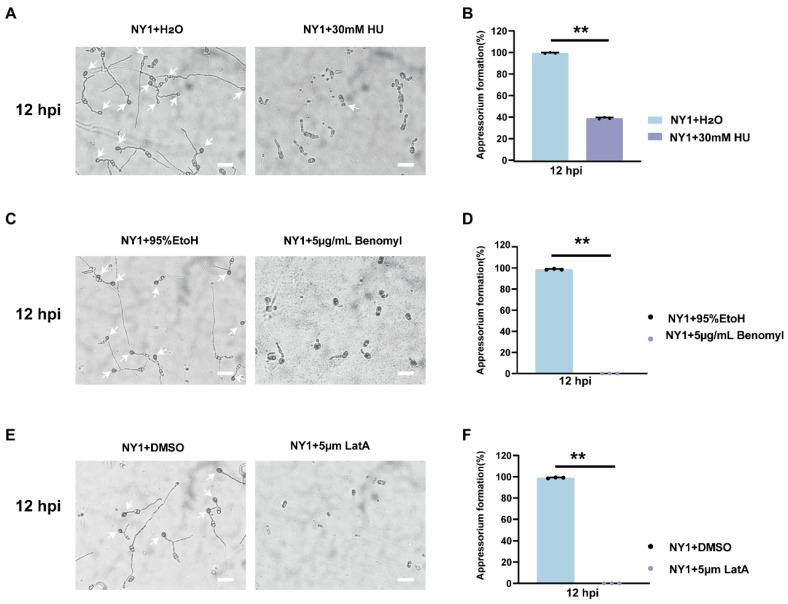
Effect of cell cycle on appressorium formation of *C. fructicola*. (**A**) Appressorium formation of *C. fructicola* treated with 30 mM HU at 12 hpi. White arrows indicate appressorium. The scale bar represents 200 µm. (**B**) Statistical analysis of conidial germination in (**A**) (n = 3 independent experiments). The above data are means ± SEs. Means with different asterisks are significantly different, as determined by the Tukey−Kramer test (** *p* < 0.01) (**C**) Appressorium formation of *C. fructicola* treated with 5 μg/mL benomyl at 12 hpi. White arrows indicate appressorium. The scale bar represents 200 µm. (**D**) Statistical analysis of conidial germination in (**C**) (n = 3 independent experiments). The above data are means ± SEs. Means with different asterisks are significantly different, as determined by the Tukey−Kramer test (** *p* < 0.01). (**E**) Appressorium formation of *C. fructicola* treated with 5 μm LatA at 12 hpi. White arrows indicate appressorium. The scale bar represents 200 µm. (**F**) Statistical analysis of conidia germination in (**E**) (n = 3 independent experiments). The above data are means ± SEs. Means with different asterisks are significantly different, as determined by the Tukey−Kramer test (** *p* < 0.01).

To investigate whether appressorium formation in *C. fructicola* is cell cycle-regulated, HU, benomyl, and LatA were separately added to the conidial suspension at 0 h. At 12 hpi, the appressorium formation rate of HU-treated NY1 dropped to 38%, and the formed appressoria were malformed (Figure 9A,B). However, benomyl and LatA treatments completely abolished appressorium formation (Figure 9C–F). These findings indicate that normal appressorium differentiation in *C. fructicola* was strictly dependent on an intact cell cycle.

### 3.11. Examination of the Roles of Cell Cycle Progression and Cytoskeletal Integrity in Early C. fructicola Infection

**Figure 10:**
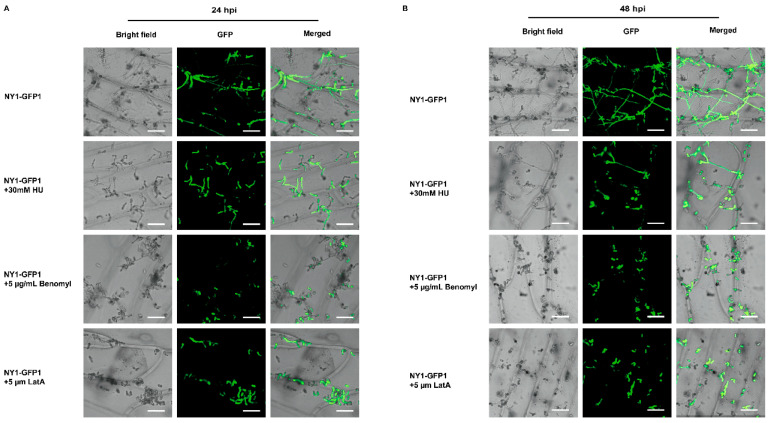
Effects of cell cycle inhibitors on early intercellular expansion of *C. fructicola* conidia in onion epidermal cells. (**A**) Effects of cell cycle inhibitors on intercellular expansion of *C. fructicola* conidia in onion epidermal cells at 24 hpi. The scale bar represents 50 µm (**B**) Effects of cell cycle inhibitors on intercellular expansion of *C. fructicola* conidia in onion epidermal cells at 48 hpi. The scale bar represents 50 µm.

To further explore the cell cycle’s role in *C*. *fructicola* physiology during early plant infection, HU, benomyl, and LatA were separately added to GFP-tagged conidial suspensions. At 24 hpi, untreated control conidia germinated, formed primary hyphae and expanded intercellularly (Figure 10A). In contrast, HU-treated conidia germinated but failed to expand intercellularly, while benomyl- and LatA-treated conidia exhibited no primary hyphae (Figure 10A). Subsequently, at 48 hpi, control hyphae continued to expand intercellularly and produced new conidia (Figure 10B). Meanwhile, only a small number of primary hyphae were formed in HU-treated conidia, and their expansion area was significantly smaller than that of the control group at 48 hpi (Figure 10B). Similarly, conidia treated with benomyl and LatA still exhibited almost complete germination failure, with severe impairment of intercellular expansion. (Figure 10B). These results indicate that cell cycle progression and cytoskeletal integrity are essential in *C. fructicola* for key infection processes, including conidial germination, appressorium formation, primary hyphal formation, and intercellular expansion.

## 4. Discussion

There are significant differences among different plant pathogenic fungi in terms of conidia types and appressorium formation development patterns [40,41]. These differences are core evolutionary features of fungi, allowing them to adapt to specific hosts and optimize their infection strategies [42,43]. Previous research has demonstrated that *C. gloeosporioides* achieves 75% appressorium formation at 5 hpi in poplar, positively regulated by mitogen-activated protein kinase and its upstream regulators CgSte50, CgSte11, and CgSte7 [44]. Maize-infecting *C. graminicola* undergoes appressorium formation at 12 hpi, and its development is positively regulated by the homologous transcription factor Cgrafh1 [45]. In *Botrytis cinerea*, approximately 60% of conidia form appressorium-like structures at 8 hpi, regulated by the autophagy gene Bcatg1 [46]. Interestingly, host-derived *C. fructicola* isolates exhibit significant variations in conidial germination and appressorium formation [18]. For instance, *Camellia oleifera*-infecting *C. fructicola* exhibits a conidial germination rate of approximately 60% at 8 hpi, with its appressorium formation rate reaching 80% at 24 hpi. Additionally, the development of appressorium is regulated by the GTPase CfRab6 [47]. In this study, *C. fructicola* isolated from avocados in Pu’er, Yunnan Province, exhibited more efficient early infection characteristics. Conidial germination was essentially complete at 4 hpi, and the appressorium formation rate was over 95% at 12 hpi (Figure 6C,D). *C. fructicola* exhibited significantly faster conidial germination and appressorium formation compared with homologous strains from other host sources. However, the regulatory mechanisms of these processes in *C. fructicola* remain unclear, and further studies should be conducted via the established genetic transformation system to explore effective targets for the precision control of avocado anthracnose.

In addition, a unique developmental pattern of conidial germination was observed in this strain. This developmental pattern was highly similar to that of rubber-infecting *C. gloeosporioides* [48]. Specifically, after one end of the conidium germinated to form appressorium, the other end could continuously extend to produce new germ tubes and further develop into primary hyphae [6]. Moreover, some conidia even exhibited differentiated, forming appressoria at both ends [49]. These primary hyphae not only enhanced the binding stability between the appressorium and the host surface, but also could expand along the host surface to form new infection sites when the initial appressorium failed to penetrate the host tissue, thereby significantly improving the overall infection success rate [5,50,51]. Avocado, similar to the rubber tree, possesses a thick and tough pericarp that forms a natural barrier against pathogenic invasion. Therefore, we speculate that this unique developmental pattern of *C. fructicola* represents a consequence of its long-term coevolution with the avocado host. The thick pericarp of avocado is a robust physical defense barrier against *C. fructicola* infection; however, *C. fructicola* effectively enhances its own infection efficiency via the bipolar conidial differentiation developmental pattern. These finding provides a novel morphological perspective for in-depth studies of *C. fructicola*’s host-specific infection mechanism.

Furthermore, as the core infection mechanism for phytopathogenic fungi to breach the host’s epidermal barrier, the precise coupling of appressorium formation with the cell cycle (G_1_ → S → G_2_ → M) constitutes a key mechanism for regulating fungal pathogenicity [29,52]. Nevertheless, its regulatory mechanism exhibits distinct species specificity [26,53]. In *Candida albicans*, HU-induced S-phase arrest can switch the growth mode from yeast-like proliferation to hyphal polarized extension, but such hyphae do not express core virulence factors [54]. In *M. oryzae*, appressorium formation is strictly dependent on S-phase progression, and HU-mediated inhibition of DNA replication completely abrogates appressorium differentiation [29,55]. Similarly, the results of our study demonstrate that HU treatment significantly reduced the appressorium formation and hyphal expansion rate of *C. fructicola* (Figure 10A), whereas they were completely inhibited by benomyl and latA treatments (Figure 10B). These findings indicated that the S and M phases of the cell cycle separately regulate appressorium formation and hyphal extension in *C. fructicola*. These two phases act synergistically to collectively sustain the entire physiological process of the pathogen from conidial germination to host infection [27,54].

This species specificity also exists in *Colletotrichum* strains, where the association between cell cycle regulation patterns, appressorium development, and pathogenicity varies distinctly [53,56]. For example, in cucumber-infecting *C. orbiculare*, the Budding-uninhibited-by-benomyl-2 (Bub2)/Byr-four-alike-1 (Bfa1) dual GAP and its downstream GTPase Termination-of-M-phase-1 (Tem1) can regulate G1/S phase progression, thereby affecting appressorium development and strain infectivity [24,26,57]. In *C. higginsianum*, Bub2 can regulate G1/S phase progression and negatively regulate septum formation during appressorium development [24]. In rubber-infecting *C. gloeosporioides*, CgCFEM1 can positively regulate the cell cycle progression of conidia and germ tubes [58]. Similarly, in rubber tree-infecting *C. gloeosporioides*, CgNOXB and CgNOXR control the specific remodeling of the F-actin cytoskeleton at the hyphal tips and appressoria, and positively regulate fungal pathogenicity [59].

Although there are some studies on cell cycle regulation in the genus *Colletotrichum*, research on *C. fructicola* is still relatively scarce. This study further revealed that the conidial germination stage in avocado-infecting *C. fructicola* mainly depends on the dynamic regulation of actin filaments and tubulin (Figure 8). In contrast, appressorium formation and hyphal expansion require a complete cell cycle (Figure 9). Based on the above findings, it has been clarified that actin filaments and tubulin are the core targets for the cell cycle regulation and pathogenicity of *C. fructicola*. In the future, a stable genetic system, combined with the molecular mechanism of cell cycle regulation, can be established to explore the key genes regulating the cell cycle of *C. fructicola* in avocados. This will enable the development of new fungicides targeting cytoskeletal dynamic reorganization or key cell cycle molecules, and ultimately achieving precise control of avocado anthracnose.

Currently, a PEG-mediated protoplast transformation method has been successfully established for *Cordyceps javanica*, *M. oryzae*, and *Colletotrichum* spp. [60,61,62]. A key advantage of this method is its short transformation cycle and relatively straightforward operational procedure, which enables the rapid generation of transformants for diverse fungal species [63,64]. However, the transformation conditions are highly species-specific and thus require separate optimization for individual fungal species in order to achieve efficient genetic transformation [65].

A genetic transformation system for *C. fructicola* was established based on an existing PEG-CaCl_2_-mediated protoplast genetic transformation system of *C. gloeosporioides* [66]. Targeted optimization of the transformation protoplast was performed according to the specific biological characteristics of this strain. Given the greater conidial production rate of *C. fructicola* compared to *C. gloeosporioides*, residual conidia may interfere with protoplast preparation and the subsequent transformation efficiency. The culture conditions for fresh *C. fructicola* mycelia were optimized before enzymatic hydrolysis. Specifically, the original conditions (28 °C, 150 rpm for 14 h) were adjusted to 28 °C and 80 rpm for 10 h. Meanwhile, the mycelia were rinsed repeatedly with sterile water to remove residual conidia. This treatment effectively eliminated the adverse impact of conidia on transformation efficiency. Furthermore, different phytopathogenic fungi exhibit distinct sensitivities to hygromycin B. Through a hygromycin B resistance gradient screening assay, 40 mg/L was identified as the optimal screening concentration for *C. fructicola* (Figure S2A). This concentration not only ensures screening efficiency but also prevents the inhibition of transformant growth at excessively high antibiotic concentrations. Furthermore, in our study, protoplast transformation was performed using 2 μg of genomic DNA as the template, and more than 30 transformants were obtained per transformation. Observation under a handheld UV lamp showed that the mycelia of more than 25 transformants emitted green fluorescence, indicating the initial expression of the GFP gene. To ensure the validity of the transformants, we further selected 8 transformants with the strongest fluorescence intensity for PCR verification. The results showed that specific bands of the GFP gene were detected in all 8 transformants, confirming that the GFP gene had been successfully integrated into the genome of *C. fructicola* (Figure S2B). The above findings confirm that a PEG-CaCl_2_-mediated genetic transformation system for the protoplasts of avocado-derived *C. fructicola* was successfully established in this study. This system lays a critical technical foundation for subsequent gene knockout-based functional analysis and investigations of *C. fructicola* infection mechanisms.

## 5. Conclusions

In this study, we isolated and purified *C. fructicola* from Pu’er avocados. An efficient genetic transformation system was established for this pathogen, and its core biological function, unique infection phenotype and cell cycle regulatory mechanism were revealed. This study’s findings not only establish a firm foundation for postharvest control strategies against *C. fructicola* but also provide robust technical and theoretical support for the sustainable development of the avocado industry.

## Figures and Tables

**Table 1:** GenBank accession numbers used for phylogenetic analysis of *C. fructicola*.

Species and Strain	Locality/Host	GenBank Accession Number		
		*ITS*	*ACT*	*GAPDH*
***Colletotrichum fructicola***				
**NY1**	**China/*Persea americana***	**PX659873**	**PX683720**	**PX683721**
SWFU 12	China/*Persea americana*	PQ866913	PQ997936	PQ997936
DS-2	China/*Pyrus bretschneideri*	KC410780	KC410781	KC410783
TJ01	China/*Phoebe sheareri*	MZ088144	MZ133608	MZ133607
Y18-3	China/Unknown	ON619598	ON638735	ON773436
***Colletotrichum salsolae***				
ICMP:19051	New Zealand/*Salsola tragus*	JX010242	JX009562	JX009916
CBS:119296	New Zealand/*Glycine max*	JX010241	JX009559	JX009917
***Colletotrichum queenslandicum***				
RHCOL1	USA/*Nephelium lappaceum*	KT372377	KT372386	KT372373
RHCOL3	USA/*Nephelium lappaceum*	KT372378	KT372383	KT372374
***Colletotrichum aenigma***				
TJ325	China/*Capsicum annuum*	PV390756	PV405399	PV405405
GA050	Israel/*Persea americana*	KX620303	KX620140	KX620237
***Colletotrichum asianum***				
MFANT15	USA/*Mangifera indica* L.	PQ811885	PX715295	PX715265
ZHKUCC 21-0095	China/*Citrus maxima*	OL708418	OL855877	OL855857
***Colletotrichum magnum***				
IMI391662	Germany/Unknown	MG600771	MG600975	MG600831
CBS:57597	Germany/Unknown	MG600770	MG601037	MG600830
***Colletotrichum plurivorum***				
24MGC68-2	China/*Mangifera indica* L.	PQ738182	PQ803202	PQ807008
JD52	China/*Vigna unguiculata*	OM857970	OM960721	OM960709
BLH3-1	China/*Passiflora edulis*	PP690820	PQ007627	PQ007587
***Colletotrichum vittalense***				
CBS 18182	Germany/Unknown	MG600734	MG600940	MG600796
CBS 12625	Germany/Unknown	MG600735	MG600941	MG600797
***Colletotrichum soja***				
XTJ2	China/*Panax ginseng*	MW048745	MW053384	MW053381
LJ2-11	China/Unknown	PP396942	PP480840	PP480952
***Colletotrichum orchidearum***				
CBS 136877	Germany/Unknown	MG600739	MG600945	MG600801
LZJZ6	China/*Philodendron*	MK796541	MK796548	MK796574
***Colletotrichum musicola***				
UACH290	Mexico/*Colocasia esculenta*	MK882586	MK882589	MK882587
COUFPI235	Brazil/*Mucuna pruriens*	PQ304842	PQ610128	PQ610146
DL87	China/*Litchi chinensis*	OR461234	OR456046	OR455970
***Mucuna pruriens***				
CBS:869.96	USA/Unknown	JQ005780	JQ005843	JX546612

Note: The bold text denotes strain NY1 isolated from diseased avocado in this study, and its GenBank accession numbers are PX659873 (ITS), PX683720 (ACT), and PX683721 (GAPDH).

## Data Availability

The original contributions presented in this study are included in the article/Supplementary Materials. Further inquiries can be directed to the corresponding author.

## Supplementary Materials

The following supporting information can be downloaded at: https://www.mdpi.com/article/10.3390/foods15081295/s1, Figure S1. DNA extraction and sequences acquisition. Figure S2. Resistance of *C. fructicola* to Hygromycin B. Figure S3. No difference in mycelial growth between GFP-tagged transformants and NY1. Figure S4. No difference in conidial yield was observed between GFP-tagged transformants and NY1. Figure S5. No difference in pathogenicity between GFP-tagged transformants and NY1. Figure S6. Effects of different concentrations of HU, benomyl and Lat A on conidial germination of *C. fructicola*. Table S1. Primers used for the study. Table S2. Formulations of the different culture media.
