# Supplementary material for: Elucidation of the Biological Function and Early-Infection Cell Cycle Regulatory Mechanism of Avocado-Infecting Colletotrichum fructicola
Source: Foods. 2026 Apr 9;15(8):1295. doi: 10.3390/foods15081295 (PMC13115315; doi:10.3390/foods15081295)
Supplement: Supplementary file 1 [file foods-15-01295-s001.zip › foods-4193277-supplementary.pdf]

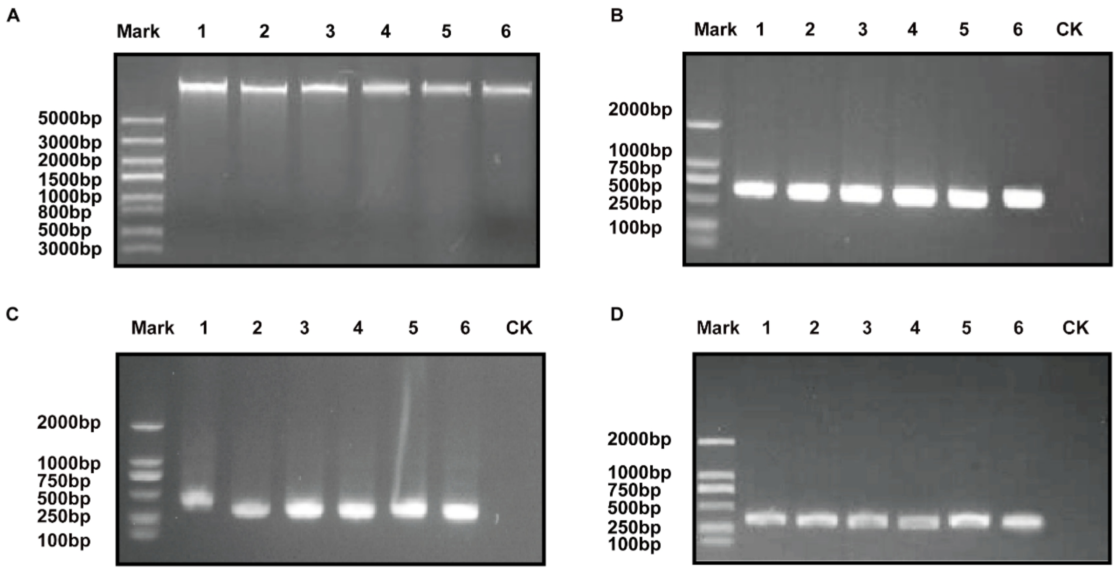

Figure S1. DNA extraction and sequences acquisition. (A) Genomic DNA *Colletotrichum fructicola*. 1-6: DNA from *C. fructicola*. (B) Diagram of the internal transcribed spacer region. 1-6: DNA from *C. fructicola* as templates, CK: dd H_2O as template. (C) Diagram of the partial sequences of actin, 1-6: DNA from *C. fructicola* as templates, CK: dd H_2O as template. (D) Diagram of and glyceraldehyde-3-phosphate dehydrogenase genes of *Colletotrichum fructicola*. 1-6: DNA from *C. fructicola* as templates, CK: dd H_2O as template.

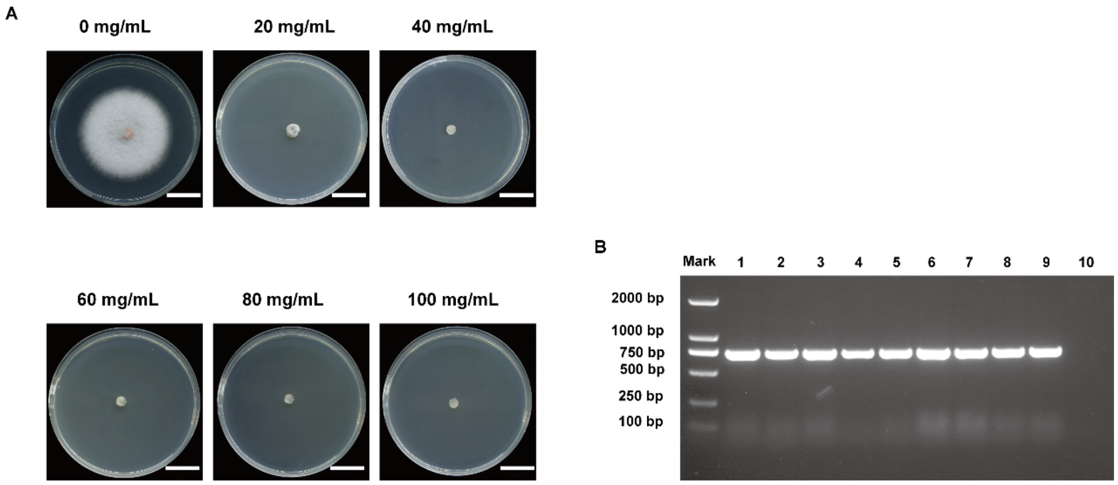

Figure S2. Resistance of *C. fructicola* to Hygromycin B. (A) Growth of *C. fructicola* on hygromycin-resistant plates with concentrations ranging from 0 to 100 mg/L. Scale bar represents 1 cm. (B) Diagram of GFP-tagged *C. fructicola* transformants. Using genomic DNA from the GFP-tagged transformants as the template, the target fragment was amplified with the GFP/F and GFP/R primers, and the amplified product was detected by agarose gel electrophoresis. 1-8: DNA from GFP-tagged transformants as templates, 9: SK1044 as template, 10: DNA from WT as template.

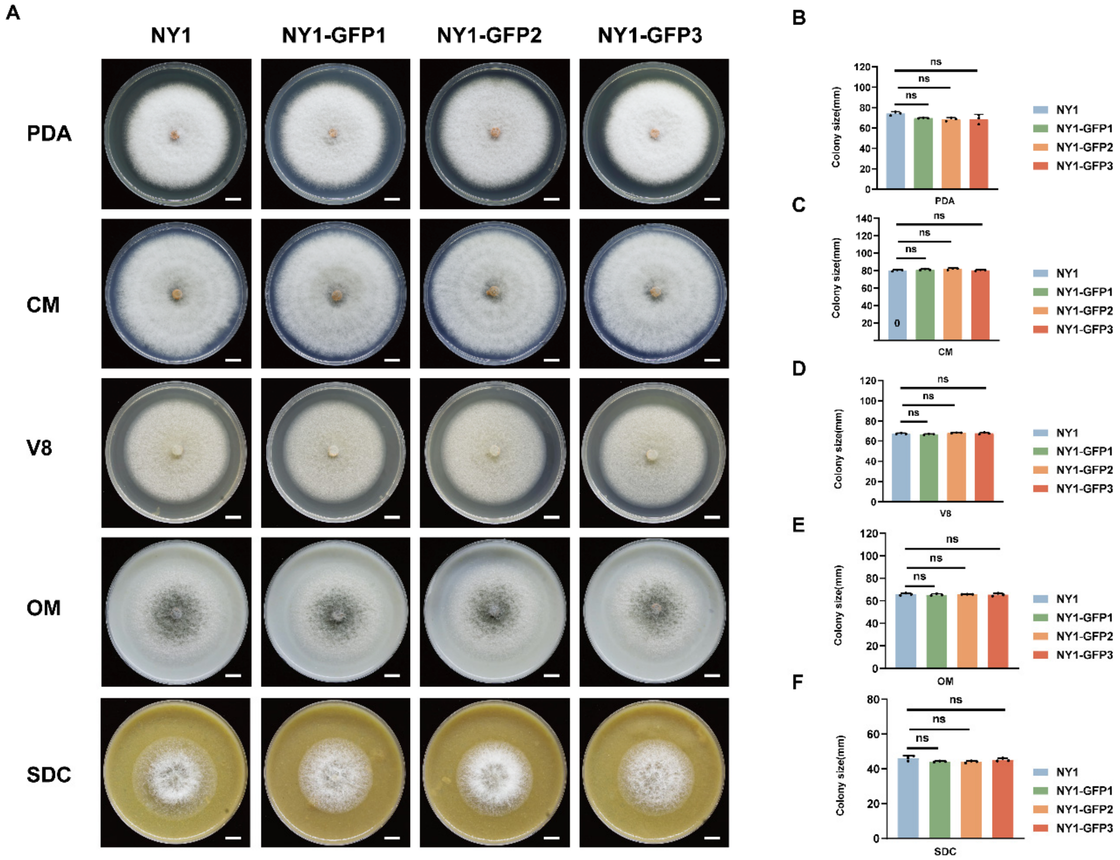

Figure S3. No difference in mycelial growth between GFP-tagged transformants and NY1. (A) Colony formation of GFP-tagged transformants and WT on PDA, CM, V8, SDC, and OM media at 6 dpi. Scale bar represents 1cm. (B-F) Statistical analysis of the colony diameters in five culture media (n = 3 independent experiments). The above data are means ± SEs. Means with different asterisks are significantly different, as determined by the Tukey−Kramer test. ns, not significant.

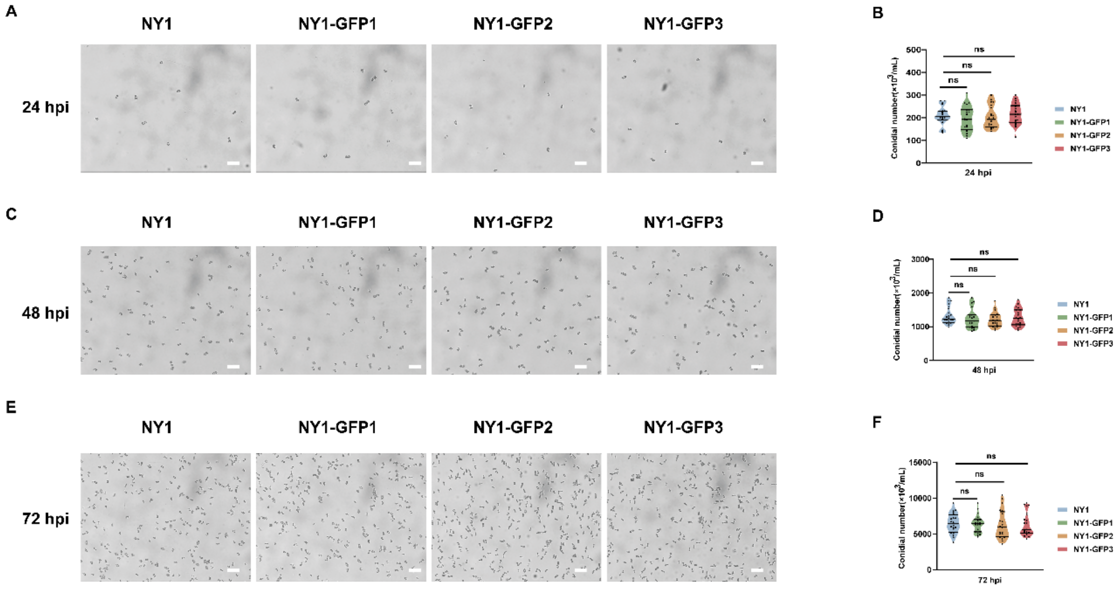

Figure S4. No difference in conidial yield was observed between GFP-tagged transformants and NY1. (A) Conidial yield under microscopic observation in CM of NY1 and GFP-tagged transformants at 24 hpi. (B) Statistical analysis of conidial yield in (A). (C) Conidial yield under microscopic observation in CM of WT and GFP-tagged transformants at 48 hpi. (D) Statistical analysis of conidial yield in (C). (E) Conidial yield under microscopic observation in CM of WT and GFP-tagged transformants at 72 hpi. (F) Statistical analysis of conidial yield in (E). All scale bar represents 200 μm in Fig S4 (n = 3 independent experiments). The above data are means ± SEs. Means with different asterisks are significantly different, as determined by the Tukey−Kramer test. ns, not significant.

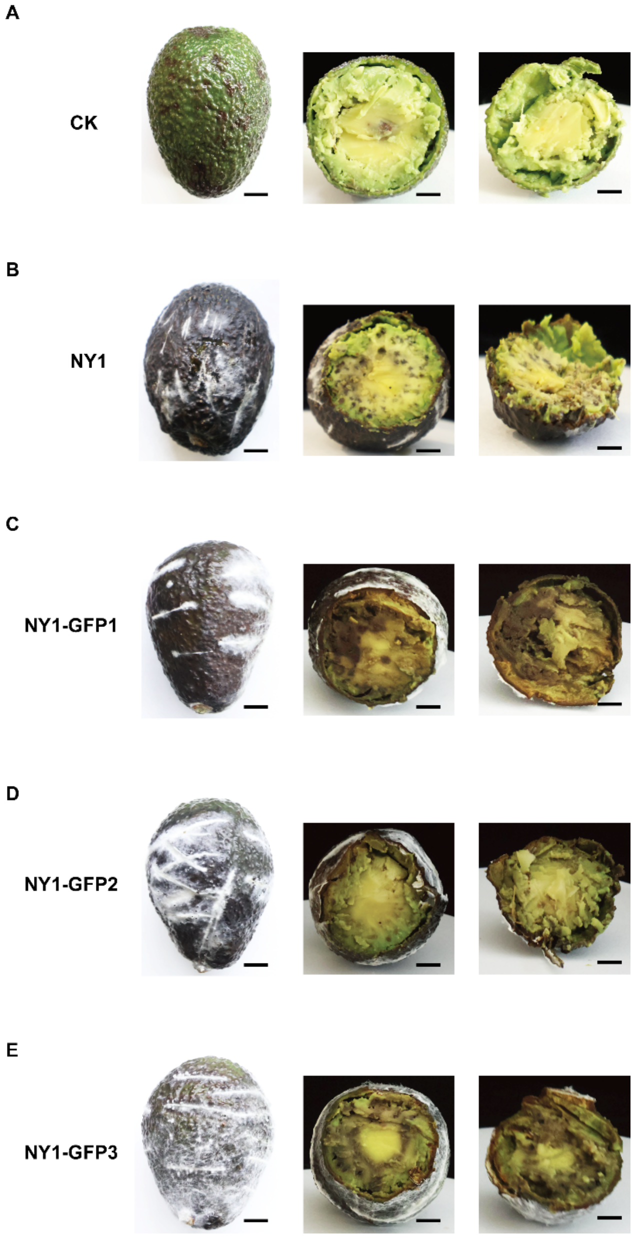

Figure S5. No difference in pathogenicity between GFP-tagged transformants and NY1. (A) Avocado fruits and their longitudinal sections inoculated with sterile water at 10 dpi. (B) Avocado fruits and their longitudinal

sections inoculated with WT at 10 dpi. (C-E) Avocado fruits and their longitudinal sections inoculated with GFP-tagged transformants at 10 dpi. All scale bar represents 1 cm in Fig S5.

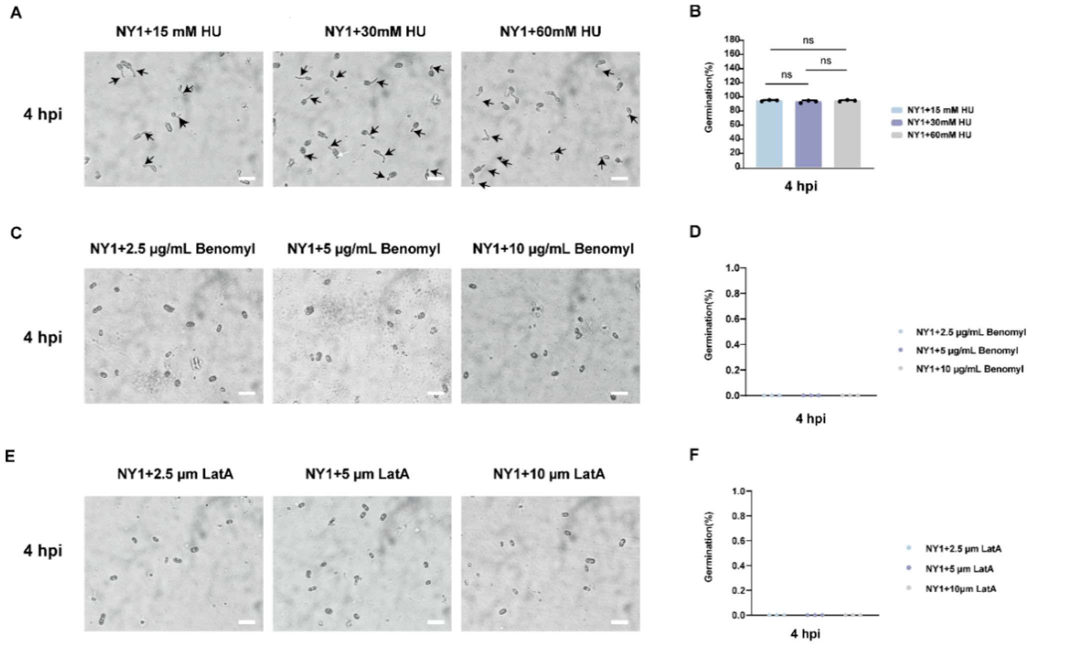

Figure S6. Effects of different concentrations of HU, benomyl and Lat A on conidial germination of *C. fructicola*. (A) Conidial germination of *C. fructicola* treated with different concentrations of HU at 4 hpi. White arrows pointed to germ tubes. Scale bar represents 200 μm. (B) Statistical analysis of conidia germination in (A). (C) Conidial germination of *C. fructicola* treated with different concentrations of benomyl at 4 hpi. Scale bar represents 200 μm. (D) Statistical analysis of conidia germination in (C). (E) Conidial germination of *C. fructicola* treated with different concentrations of Lat A at 4 hpi. Scale bar represents 200 μm. (F) Statistical analysis of conidia germination in (E) (n = 3 independent experiments). The above data are means ± SEs. Means with different asterisks are significantly different, as determined by the Tukey−Kramer test. ns, not significant.

Table S1. Primers used for the study

Primers	Primers sequence (5'-3')
ITS4	TCCTCCGCTTATTGATATGC
ITS5	GGAAGTAAAAGTCGTAACAAGG
ACT512F	ATGTGCAAGGCCGGTTTCGC
ACT783R	TACGAGTCCTTCTGGCCCAT
GDF	GCCGTCAACGACCCCTTCATTGA
Primers	Primers sequence (5'-3')

GDR	GGGTGGAGTCGTACTTGAGCATGT
GFPF	ATGGTGAGCAAGGGCGAGG
GFPR	CTACTTGTACAGCTCGTCCAT

Table S2. Formulations of the different culture media

Medium name	Formulation	Sterilization condition
PDA	46 g/L, Antibiotic-free dextrose potato medium prepared by Solarbio was purchased.	121°C, 20 min
TB3	3 g/L yeast extract, 3 g/L casamino acids, 200 g/L sucrose, and 12% agar	121°C, 20 min
OM	50 g/L oatmeal, dissolved with dd H_2O, filtered through a layer of gauze, added 15% agar	121°C, 20 min
V8	100 ml V8, 0.2 g/L $CaCO_3$, 15% agar	121°C, 20 min
SDC	100 g/L of rice straw, boiled for 20 min, filtered through two layers of gauze, discarded the residue, added 40 g/L of corn flour and 15% agar	121°C, 20 min
CM	50 mL 20×Nitrate Salts, 1 mL Trace Elements, 10 g/L D-Glucose, 2 g/L Peptone, 1 g/L Yeast Extract, 1 g/L Casamino Acids, 1 mL Vitamin Solution, 15% Agar; 20×Nitrate Salts: 120 g/L $NaNO_3$, 10.4 g/L KCl, 10.4 g/L $MgSO_4 \cdot 7H_2O$, 30.4 g/L KH_2PO_4, Store at 4 °C; 1000×Trace Elements: 2.2 g/L $ZnSO_4 \cdot 7H_2O$, 1.1 g/L H_3BO_3, 0.5 g/L $MnCl_2 \cdot 4\ H_2O$, 0.5 g/L $FeSO_4 \cdot 7H_2O$, 0.17 g/L $CoCl_2 \cdot 6H_2O$, 0.16 g/L $CuSO_4 \cdot 5\ H_2O$, 0.15 g/L $Na_2MnO_4 \cdot 2H_2O$, 5 g/L Na_4EDTA, Store at 4°C; Vitamin Solution: 0.01 g/L Biotin, 0.01 g/L Pyridoxin, 0.01 g/L Thiamin, 0.01 g/L Riboflavin, 0.01 g/L PABA (Para-aminobenzoic acid), 0.01 g/L Nicotinic Acid, Store at 4 °C in the dark.	121°C, 15 min, NaOH to pH to 6.5
